# 儿童慢性髓性白血病急变期临床特征及预后的研究

**DOI:** 10.3760/cma.j.cn121090-20240130-00045

**Published:** 2024-10

**Authors:** 方圆 郑, 爱东 陆, 月萍 贾, 英熹 左, 慧敏 曾, 倩 江, 乐萍 张

**Affiliations:** 1 北京大学人民医院儿科，北京 100044 Peking University People's Hospital, Department of Pediatrics, Beijing 100044, China; 2 北京大学人民医院血液科、北京大学血液病研究所、国家血液系统疾病临床医学研究中心，北京 100044 Peking University People's Hospital, Peking University Institute of Hematology, National Clinical Research Center for Hematologic Disease, Beijing 100044, China

**Keywords:** 白血病，髓性，慢性, 急变期, 儿童, Leukemia, myeloid, chronic, Blast phase, Children

## Abstract

**目的:**

探讨儿童慢性髓性白血病急变期（CML-BP）患者的临床特征及生存结局。

**方法:**

回顾性分析北京大学人民医院2008年1月至2022年11月应用酪氨酸激酶抑制剂（TKI）治疗的28例CML-BP患儿病例资料，分析其临床特征、治疗情况、生存结局。

**结果:**

28例CML-BP患儿中，男女比例1.15∶1，急变时中位年龄10岁，急变后中位随访时间为79个月；初诊时即为CML-BP 4例，初诊时为加速期（AP）1例，初诊断时为慢性期（CP）23例，其中有21例CML-CP未经CML-AP直接进展为CML-BP；急变类型中淋系急变占71.4％、髓系急变占25.0％、混合表型急变占3.6％；急变后行TKI联合化疗再进行造血干细胞移植（HSCT）治疗19例、单纯TKI联合HSCT治疗2例、TKI联合化疗7例。28例CML-BP患儿的5年总生存率为59.3％。

**结论:**

儿童CML-BP由CML-CP直接进展而来的比例比成人高，总体预后差。

## Abstract (en)

**Objective:**

To explore the clinical characteristics and prognosis of children with chronic myeloid leukemia in the blast phase（CML-BP）.

**Methods:**

The clinical characteristics, treatment measures, and survival outcomes of 28 children with CML-BP were analyzed in our hospital from January 2008 to November 2022.

**Results:**

The male to female ratio of the 28 children with CML-BP was 1.15∶1. The median age of diagnosis of CML-BP was 10 years, and the median follow-up time was 79 months. During the diagnosis of CML, four children were in the BP, one was in the accelerated phase（AP）and 23 children were in the chronic phase（CP）. Among the 23 children with CML-CP, 75% had progressed directly from CP to BP without experiencing the AP. Among the children diagnosed with CML-BP, 71.4% were classified as chronic myeloid leukemia lymphoid blast phase（CML-LBP）, 25.0% belonged to the chronic myeloid leukemia myeloid blast phase（CML-MBP）, and 3.6% belonged to the chronic myeloid leukemia mixed phenotype acute leukemia（CML-MPAL）. Treatment with hemaopoietic stem cell transplantation（HSCT）after tyosine kinase inhibitor（TKI）combined with chemotherapy was administered to 19 children, two children received HSCT after TKI alone, and seven children received TKI combined with chemotherapy but without HSCT. The 5-year overall survival of the 28 children with CML-BP was 59.3%.

**Conclusion:**

The direct progression of BP from CP is greater in children with CML-BP compared with adults, and the overall prognosis of children with CML-BP is poor.

慢性髓性白血病（CML）是一种起源于造血干细胞的骨髓增殖性肿瘤，自然病程包括慢性期（CP）、加速期（AP）及急变期（BP）[1]，其在儿童中为罕见病，年发病率为0.6～1.2/100万儿童[2]。随着酪氨酸激酶抑制剂（TKI）的研发以及CML诊疗的规范化，儿童CML的长期生存情况明显改善，但有部分患儿以CML-BP起病或对TKI耐药等因素进展为CML-BP，该阶段作为CML疾病的终末期，预后差，治疗棘手[3]–[5]。本研究回顾性分析我院收治的28例儿童CML-BP患者的临床特征、治疗情况、生存结局等，旨在为儿童CML-BP临床管理的进一步完善提供依据。

## 对象与方法

1. 研究对象：回顾性分析2008年1月至2022年11月于北京大学人民医院就诊的儿童CML-BP患者的临床数据。纳入标准：①符合欧洲白血病网（ELN）2020指南[1]CML-BP诊断标准；②CML-BP诊断年龄<18岁。排除标准：未应用TKI药物治疗。

本研究已通过北京大学人民医院伦理委员会批准（2022PHB247-001），本研究中所有患儿的监护人在治疗前均被告知治疗方案，并签署知情同意书。

2. 治疗方法：参照CML诊疗指南[1],[3]，在诊断CML-BP后，应用TKI作为一线治疗，并根据免疫表型判断急变类型（髓系、淋系），辅以相应的诱导化疗，并定期监测，条件允许时尽快行造血干细胞移植（HSCT）治疗。

具体为：①TKI药物选择：考虑患儿个体化差异，从疾病诊断风险、经济承受能力、ABL1激酶区突变等多个因素来选择TKI药物。②诱导化疗方案：淋系急变（Chronic myeloid leukemia lymphoid blast phase，CML-LBP）者主要为地塞米松、长春碱类、蒽环类、门冬酰胺酶、环磷酰胺等。髓系急变（Chronic myeloid leukemia myeloid blast phase，CML-MBP）者主要为阿糖胞苷、高三尖杉酯碱、蒽环类或亚砷酸等。混合表型白血病急变（Chronic myeloid leukemia mixed phenotype acute leukemia, CML-MPAL）者主要参考CML-LBP的方案。③HSCT方案：依据患儿与供者骨髓人类白细胞抗原（HLA）配型结果等进行半相合HSCT或全相合HSCT。

3. 疾病定义：CML的诊断及分期标准、治疗反应参照ELN 2020指南[1]，其中CML-BP定义为符合至少1项指标：①骨髓和（或）外周血原始细胞≥30％；②髓外原始细胞浸润（除脾脏外）。CML治疗反应中，完全血液学反应（CHR）需满足以下5条：①PLT<450×10^9^/L；②WBC<10×10^9^/L；③嗜碱性粒细胞<5％；④外周血中无髓性不成熟细胞；⑤无疾病相关症状、体征，可触及的脾肿大消失。完全细胞遗传学反应（CCyR）是指Ph染色体阳性细胞0％，主要分子学反应（MMR）是指BCR::ABL1/ABL1^IS^ ≤0.1％。

急变后诊断及治疗效果参照张之南等主编的《血液病诊断及疗效标准》，其中疗效评估：①完全缓解（CR）：骨髓原始细胞<5％，外周血白细胞分类无白血病细胞，具有再生造血功能（PLT>100×10^9^/L，中性粒细胞>1×10^9^/L）且无局部白血病浸润。②复发：实现CR后骨髓原始细胞再次≥5％，外周血中再次出现白血病细胞或髓外任何部位出现白血病细胞浸润。

4. 随访：末次随访时间为2022年12月31日，对于失访患儿截至对该患儿末次随访时间。总生存（OS）期指从诊断CML-BP到任何原因死亡的时间或末次随访时间。

5. 统计学处理：疾病特征采用描述性统计分析，连续变量描述为*M*（范围），分类变量描述为例数（百分比）。使用Kaplan-Meier方法估计生存函数。统计学分析采用SPSS19.0及R软件。

## 结果

1. 患者基本情况：本研究共获得29例2008年1月至2022年11月于我院诊治的诊断CML-BP、年龄<18周岁患者的临床资料，排除1例未应用TKI药物治疗，共有28例CML-BP患儿纳入研究。男15例，女13例。

2. 初诊CML时临床特征及治疗情况：初诊CML时中位年龄10（2～16）岁，分期为CML-CP 23例（82.1％），CML-AP 1例（3.6％），CML-BP 4例（14.3％）。WBC、HGB含量、PLT详见表1。

**表1:** 28例慢性髓性白血病（CML）急变期患儿初诊CML时的临床特征

特征	数值
性别［例（%）］	
男	15（53.5）
女	13（46.5）
初诊年龄［岁，*M*（范围）］	10（2~16）
疾病分期［例（%）］	
慢性期	23（82.1）
加速期	1（3.6）
急变期	4（14.3）
初诊脾大［例（%）］	25（89.3）
初诊WBC［×10^9^/L，*M*（范围）］	222.1（23.7~651.2）
初诊HGB［g/L，*M*（范围）］	90（62~125）
初诊PLT［×10^9^/L，*M*（范围）］	456（52~4 534）
初诊染色体核型^a^［例（%）］	
单纯Ph染色体	18（78.3）
伴额外异常染色体	5（21.7）

**注** ^a^共有23例患儿有可分析的染色体核型资料

在初诊时为CML-CP/AP的24例患儿中，一线TKI治疗22例，分别为伊马替尼20例（包括伊马替尼联合干扰素α 2例，伊马替尼联合中药1例）、氟马替尼2例，另外，未应用TKI治疗的2例患儿分别为单纯干扰素α治疗1例，干扰素α联合阿糖胞苷化疗1例。在该24例CML-CP/AP患儿中，因药物不耐受（血液学不良反应3～4级）停药5例、因严重感染而间断停药1例、自行停药1例。该24例CML-CP/AP患者中，急变前获得MMR仅4例，达CCyR未达MMR共2例。

3. CML患儿急变时临床特征：急变时中位年龄10（3～18）岁。急变时10例（35.7％）为常规复查/体检时发现，有症状者表现为发热15例、腹胀/腹痛等消化道症状5例、乏力/面色苍白2例，部分患儿表现为≥1种症状，无出血等表现。急变时存在脾脏肿大24例（85.7％）。

急变类型中CML-LBP 20例（71.4％），均为B细胞型，其余为CML-MBP 7例（25.0％）、CML-MPAL（B系+髓系）1例（3.6％）。从初诊CML-CP进展为CML-BP中位病程为9.0（3.0～48.0）个月，其中有2例CML-CP患儿在经过CML-AP阶段后进展为CML-BP，1例初诊CML-AP患儿在1个月后进展为CML-BP，剩余21例患儿未经CML-AP阶段而直接进入CML-BP。20例CML-LBP患儿中，从初诊CML到诊断CML-LBP中位时间8.2（0～48.0）个月。7例CML-MBP患儿中，从初诊CML到诊断CML-MBP中位时间10.5（0～32.0）个月。急变时WBC、HGB含量、PLT、外周血原始细胞比例、外周血嗜碱性粒细胞比例、骨髓原始细胞比例、骨髓嗜碱性粒细胞比例详见表2。

**表2:** 28例患儿诊断CML-BP时的临床特征

特征	数值
急变时年龄［岁，*M*（范围）］	10（3~18）
急变时脾大［例（%）］	24（85.7）
急变时WBC［×10^9^/L，*M*（范围）］	69.8（1.0~403.8）
急变时HGB［g/L，*M*（范围）］	89（63~137）
急变时PLT［×10^9^/L，*M*（范围）］	108（10~1 447）
急变时外周血原始细胞［%，*M*（范围）］	40.0（0~84.0）
急变时外周血嗜碱性粒细胞［%，*M*（范围）］	1（0~22）
急变时骨髓原始+幼稚细胞［%，*M*（范围）］	62.5（21~96）
急变时骨髓嗜碱性粒细胞［%，*M*（范围）］	0（0~10）
急变类型［例（%）］	
CML-LBP	20（71.4）
CML-MBP	7（25.0）
CML-MPAL	1（3.6）
急变时染色体核型^a^［例（%）］	
单纯Ph染色体	13（52.0）
伴额外异常染色体	12（48.0）
BCR::ABL1/ABL1^IS^基因水平［%，*M*（范围）］	56.0（3.1~144.5）

**注** CML-BP：慢性髓性白血病急变期；CML-LBP：慢性髓性白血病淋系急变；CML-MBP：慢性髓性白血病髓系急变；CML-MPAL：慢性髓性白血病混合表型急变；IS：国际化标准值；^a^ 共有25例患儿有可分析的染色体核型资料

诊断CML-BP时，28例患儿中行ABL1激酶区突变检查24例，阳性11例（45.8％），阴性13例（54.2％）。28例CML-BP患儿中3例（10.7％）有髓外疾病，髓外浸润均表现为中枢神经系统白血病（CNSL）。

诊断CML-BP时，具有可分析的核型25例，均具有典型Ph染色体，其中，单纯Ph染色体为13例（52.0％），伴变异型易位染色体3例（12.0％），伴附加异常染色体9例（36.0％）（表3）。

**表3:** 12例伴额外异常染色体慢性髓性白血病急变期患儿核型分析

例号	染色体核型
1	44,XX,t(5;6)(q31;q25),−7,−9,t(9;22)(q34;q11)[10]
2	44,XY,−4,−9,t(9;22)(q34;q11),−10,−12,−24,+M1-M3[20]
3	45,XY,−7,−9,t(9;22)(q34;q11),der(22)t(9;22)[22]/46,XY[3]
4	45,XY,t(1;9;22)(p32;q34;q11)[20]
5	46,XX,add(1)(p36),t(9;22;18)(q34;q11;q21),del(16)(p11)[10]
6	46,XX,add(9)(p13),t(9;22)(q34;q11)[10]/46,XX,i(9)(q10),t(9;22)/46,XX[5]
7	46,XX,der(9)t(9;22)(q34;q11)del(p13)[10]/46,XX[10]
8	46,XX,t(9;22)(q34;q11),del(16)(q24)[20]
9	46,XY,add(1)(p36),t(9;22)[3]/46,XY,t(9;22)[17]
10	46,XY,t(6;9;22)(p21;q34;q11)[1]/46,XY[19]
11	47,XX,t(9;22)(q34;q11),+der(22)t(9;22)[20]
12	57,XX,+2,+5*2,+6,+9,t(9;22),+10,+11,+18,+19,+21,+Ph[1]/57,XX,+2,+5*2,+del(6)(q23),+8,+9,t(9;22),+11,+18,+19,+21,+Ph[1]/46,XX,t(9;22)[3]/46,XX[5]

4. 诊断CML-BP后的治疗情况及转归：28例CML-BP患儿治疗情况及转归见表4。其中，1例CML-LBP患儿（例8）在奥雷巴替尼联合VP方案（长春新碱、地塞米松）诱导化疗后，行靶向CD19的嵌合抗原受体T细胞免疫治疗，随访时等待HSCT。1例CML-MBP患儿（例9）为初诊CML-BP患儿，单纯伊马替尼治疗后3个月行HSCT。1例CML-LBP患儿（例10）为初诊CML-BP的患儿，单纯用伊马替尼治疗6个月达CCyR，后于8个月时行HSCT。1例CML-MBP患儿（例25）在达沙替尼联合HA方案（高三尖杉酯碱、阿糖胞苷）诱导化疗后未达CR，后予以奥雷巴替尼联合维奈克拉、阿扎胞苷方案化疗后已达CR，随访时等待HSCT。

**表4:** 28例患儿诊断CML-BP后的治疗情况及转归

例号	急变时年龄（岁）	性别	急变类型	点突变	初始治疗方案	诱导化疗后评估达CR	是否HSCT	转归
1	10	女	CML-MBP	1378G>A	达沙替尼+阿糖胞苷+亚砷酸	是	是	存活
2	15	女	CML-LBP	F317I、F359V	尼洛替尼+VDLD方案	是	否	死于原发病
3	3	男	CML-LBP	阴性	达沙替尼+VDLD方案	是	是	死于原发病
4	8	女	CML-MBP	阴性	伊马替尼+ADE方案	是	是	存活
5	16	男	CML-LBP	未做	达沙替尼+COP方案	否	是	死于GVHD
6	7	男	CML-LBP	阴性	普纳替尼+CODPL方案	是	是	存活
7	12	男	CML-LBP	阴性	达沙替尼+CODPL方案	是	是	存活
8	14	男	CML-LBP	T315I	奥雷巴替尼+VP方案	是	否	存活
9	13	女	CML-MBP	阴性	伊马替尼	–	是	存活
10	10	女	CML-LBP	未做	伊马替尼	–	是	存活
11	10	男	CML-MBP	阴性	伊马替尼+阿糖胞苷	是	是	存活
12	10	女	CML-LBP	阴性	伊马替尼+VDLD方案	是	是	存活
13	7	男	CML-LBP	E459K	伊马替尼+VDLD方案	是	否	死于原发病
14	15	女	CML-LBP	Y253H	尼洛替尼+CODPL方案	否	是	存活
15	13	男	CML-MBP	未做	达沙替尼+亚砷酸	死亡	否	死于原发病
16	9	男	CML-LBP	阴性	伊马替尼+CODPL方案	是	是	存活
17	18	女	CML-LBP	阴性	达沙替尼+VDLD方案	是	是	存活
18	9	女	CML-LBP	E255K	达沙替尼+VDLD方案	是	是	存活
19	9	男	CML-LBP	阴性	伊马替尼+VDLD方案	否	否	死于原发病
20	11	女	CML-LBP	Q252H	伊马替尼+CODPL方案	否	是	死于GVHD
21	16	男	CML-LBP	阴性	伊马替尼+VDCP方案	否	是	死于原发病
22	16	男	CML-LBP	T315I	伊马替尼+VP方案	否	是	死于感染性休克
23	6	女	CML-LBP	阴性	达沙替尼+CODPL方案	是	是	存活
24	4	女	CML-LBP	Y253H	达沙替尼+CODPL方案	是	是	存活
25	15	男	CML-MBP	G250E	达沙替尼+HA方案	否	否	存活
26	18	男	CML-LBP	Y253H	达沙替尼+VDLD方案	否	是	死于原发病
27	3	男	CML-MPAL（B+M）	未做	伊马替尼+VDLD方案	否	否	死于原发病
28	9	女	CML-MBP	阴性	伊马替尼+ADE方案	是	是	死于GVHD

**注** CML-BP：慢性髓性白血病急变期；CML-LBP：慢性髓性白血病淋系急变；CML-MBP：慢性髓性白血病髓系急变；CML-MPAL：慢性髓性白血病混合表型急变；CR：完全缓解；HSCT：造血干细胞移植；GVHD：移植物抗宿主病；CODPL方案：环磷酰胺+地塞米松+长春新碱类+蒽环类+门冬酰胺酶；VDLD方案：地塞米松+长春新碱类+蒽环类+门冬酰胺酶：VDCP方案：环磷酰胺+地塞米松+长春新碱类+蒽环类：VP方案：地塞米松+长春新碱类；COP方案：环磷酰胺+地塞米松+长春新碱类；ADE方案：阿糖胞苷+蒽环类+依托泊苷；HA方案：高三尖杉酯碱+阿糖胞苷；–：未进行评估

5. CML-BP患儿生存结局：28例CML-BP患儿中位OS期为22（1～180）个月，1年、2年、5年OS率分别为77.0％、64.3％、59.3％（图1）。

**图1:**
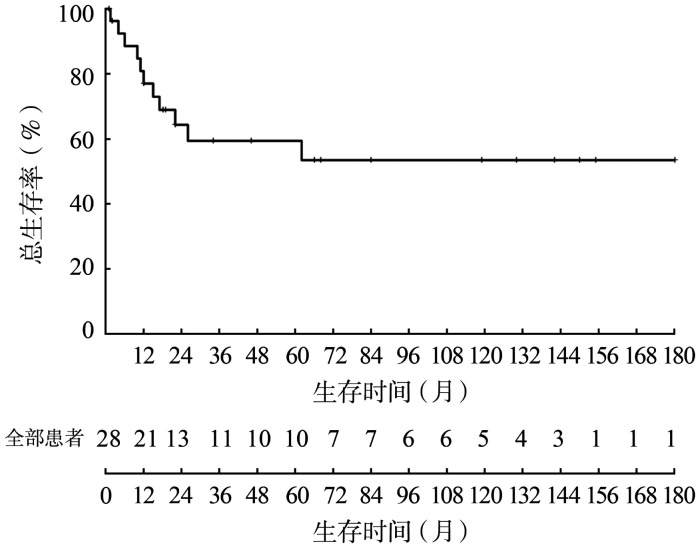
28例慢性髓性白血病急变期患儿的生存情况

## 讨论

本研究对近15年来我院诊治的CML-BP患儿的临床特征、治疗情况、生存结局进行分析，结果显示，CML-BP患儿性别比例、年龄分布、急变类型以CML-LBP为主等的特征与国外研究相似[3],[6]–[8]。本研究发现儿童CML-BP由CP未经AP直接进展而来者较成人所占比例高，CML-BP预后差，5年OS率为59.3％。

在成人，CML急变大多是逐渐发生的，由CP进展为AP更进一步进展到BP。50％～80％患者进入BP前有明确的AP，仅有少数患者原始细胞在数日至数周之内迅速增长而短期内进展到BP[5]。在Kantarjian等[9]研究的1 093例成人CML患者中，出现急变183例，其中46例为突发急变，仅占25％。本研究的28例CML-BP患儿中，有明确AP史者占10.7％，另外，初诊CML-BP占14.2％，由CML-CP短期内直接进展为CML-BP占75％，提示儿童CML-BP由CP未经AP直接进展而来者较成人所占比例高。分析原因，可能与儿童CML-LBP所占比例较大有关。结合成人CML研究[10]以及儿童CML研究[6]均发现淋系急变比髓系急变发生更突然，而儿童CML-BP中淋系急变占比较大，此可能一定程度上解释儿童CML较成人CML进展快的原因。此外，儿童CML-BP大部分不经过AP而直接由CP进展而来，也反映儿童CML较成人CML更有侵袭性、进展快的特点。

在由CML-CP进展为CML-BP的因素中，早期获得深度分子学反应是降低疾病进展风险的因素[11]。本研究中在初诊为CML-CP/AP的24例患者急变前获得MMR仅占16％，达CCyR未达MMR仅占8.3％。提示在CML-CP/AP患者中，TKI治疗后效果评估的重要性，以及警惕在反应欠佳时有疾病进展的可能性。国外文献中，存在主要途径附加染色体异常［如+8、+Ph、i（17q）、+19等］也是疾病进展的常见原因[11]，但因本研究样本量少，未进行相关分析。此外，在TKI治疗的CML患儿中，治疗依从性差是导致疗效不佳、疾病进展的常见原因之一。本研究在初诊为CML-CP/AP的24例患者中，急变前因药物毒性停药、自愿停药者高达29.2％。提示在CML儿童的临床管理中，关注提高治疗依从性、合理选择靶向药物以减轻药物不良反应的重要性，尽量减少因反复停药而引起疾病进展。

成人CML-BP中有4％～16％患者出现髓外原始细胞浸润，最常见的部位为淋巴结、CNS、骨骼系统、肌肉、皮肤等[12]。髓外急变时可合并骨髓内急变，亦有部分患者在骨髓处于完全缓解期、CP或者AP而发现髓外急变，一般此类患者在1～12个月后亦会出现骨髓内急变[13]。Millot等[8]研究的17例儿童CML-BP患者中，6例有髓外浸润，分别为皮肤浸润（1例）、淋巴结浸润（1例）、髓样肉瘤（2例）和CNS浸润（2例）。本研究中有3例（10.7％）有髓外疾病，髓外浸润均表现为髓内急变合并CNSL。因此，对于CML患儿，即使是慢性期，如出现淋巴结肿大、皮肤包块、视力下降、听力下降或其他症状时，要警惕髓外急变可能。

目前CML-BP的治疗方案仍在不断探索中。儿童CML相关专家共识及疾病管理建议[3],[14]–[15]，对于所有初诊时为CML-BP或在TKI治疗中进展为CML-BP的患儿，都应在TKI或联合化疗回到CML-CP后尽快进行HSCT。TKI的选择多建议依据ABL1激酶区突变检测结果选择TKI。关于所选择的诱导化疗的最佳方案尚未确定，目前多建议在TKI靶向治疗基础上加用ALL或AML方案进行化疗[3],[11],[16]–[17]，对于初诊时为BP患儿且不能适用强化疗者，部分专家认为也可以单独使用TKI，但需要更密切的检测[11]。本研究中急变后行TKI联合化疗再进行HSCT治疗19例，随访时有13例长期存活，一定程度提示HSCT可改善预后。

总之，本研究发现儿童CML-BP由CML-CP直接进展而来的比例比成人高，总体预后差。但由于儿童CML-BP发病率极低，临床上罕见，故单中心回顾性研究的病例数较少，部分预后情况以描述性统计分析为主，此外，本中心在TKI联合化疗的治疗中，传统化疗方案的选择上更为个体化，统计学上无法得出何种方案CR率更高。因此，未来需要多中心合作，累积更多的病例来进行更深入的分析、总结。
